# Supplementary material for: Digital aberration correction for enhanced thick tissue imaging exploiting aberration matrix and tilt-tilt correlation from the optical memory effect
Source: Nat Commun. 2025 Feb 16;16:1685. doi: 10.1038/s41467-025-56865-z (PMC11830826; doi:10.1038/s41467-025-56865-z)
Supplement: Supplementary file 1 — Supplementary Information [file 41467_2025_56865_MOESM1_ESM.pdf]

Supplementary Information: Digital aberration correction for enhanced thick tissue imaging exploiting aberration matrix and tilt-tilt correlation from the optical memory effect

ChulMin Oh[1,2], Herve Hugonnet[1,2], Moosung Lee[1,2,3,4], YongKeun Park[1,2,5,*]

[1]*Department of Physics, Korea Advanced Institute of Science and Technology, Daejeon 34141, Republic of Korea*

[2]*KAIST Institute for Health Science and Technology, KAIST, Daejeon 34141, Republic of Korea*

[3]*Institute for Functional Matter and Quantum Technologies, Universität Stuttgart, 70569 Stuttgart, Germany*

[4]*Current affiliation: Center for Integrated Quantum Science and Technology (IQST), University of Stuttgart, 70569 Stuttgart, Germany*

[5]*Tomocube, Inc., Daejeon 34051, Republic of Korea*

[*]*Correspondence to: yk.park@kaist.ac.kr (Y.P.)*

1. Application to time-gated reflection imaging in biological tissues

1.1. Configuration of the simulation

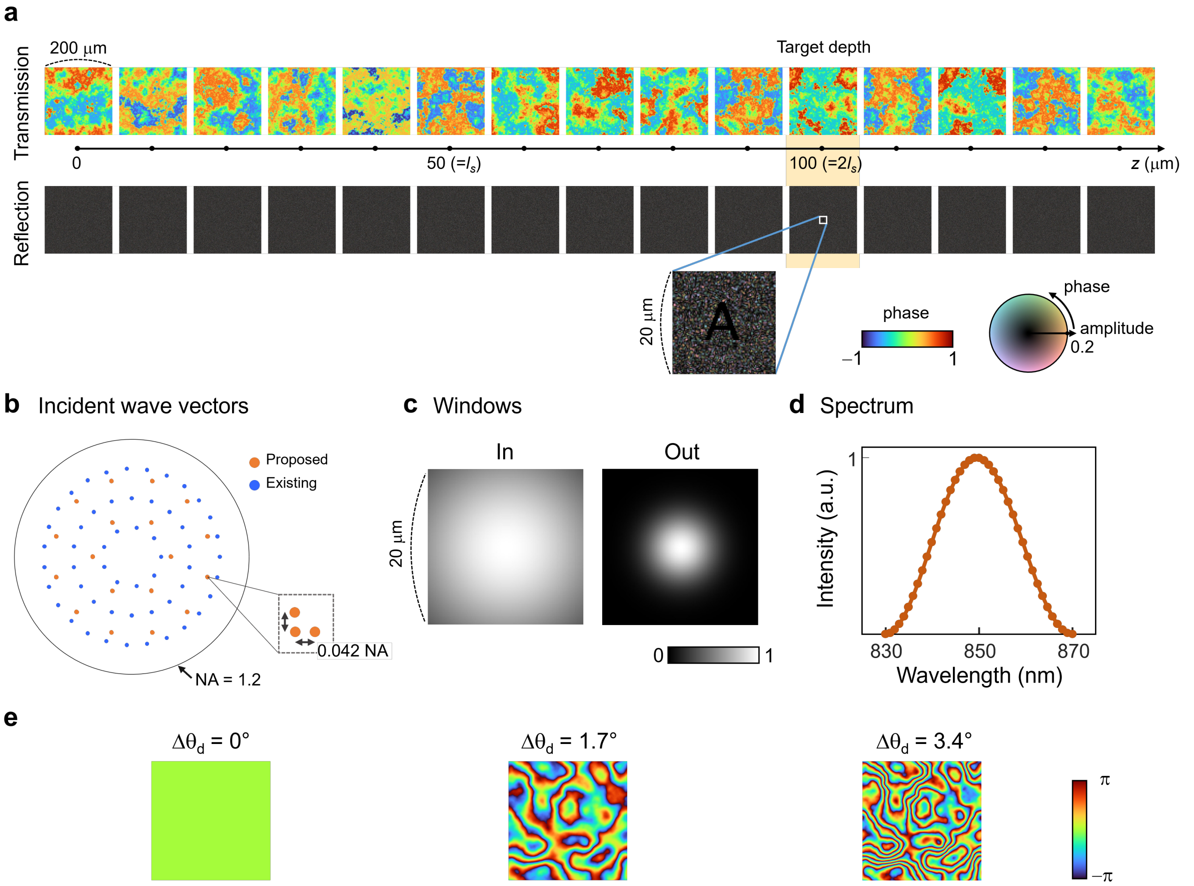

Supplementary Figure 1: Detailed configuration of the simulation. a, Transmission and reflection coefficient of 15 layers to simulate 150 μm-thick biological tissue. Image of letter 'A' is embedded in the target plane. The amplitude of transmission coefficients, obmitted from the figure, are set such that the sum of the transmittance and reflectance is unity in each position. **b**, Configuration of 54 incident wave vectors for the proposed (orange) and existing computational (blue) AO methods. **c**, Window functions for incoming and outgoing waves. **d**, Spectrum of the light source used in the simulation. Here, the size of the window function (σ = 10 μm) for the incoming waves is matched to the target FOV, which is approximately the largest PSF size in the simulation. In contrast, those for the outgoing waves (σ = 3 μm) are adjusted to maximize the reliable convergence of the existing computational methods. **e**, Phase functions with varying mean diffraction angles ($\Delta\theta_d$) were applied for cases of no surface deflection (left), mild deflection (middle), and significant surface deflection (right). Under practical conditions, light deflects at the interface between tissue and its surrounding medium, following the tissue's surface

profile. This deflection can introduce substantial aberration, particularly when there is a large refractive index mismatch at the interface. To model this boundary-induced light deflection, smooth phase functions were incorporated into the transmission coefficient of the first layer.

1.2. Effect of iterative windowing combined with the aberration matrix

In Supplementary Fig. 2, we present a visual representation of the effect of iterative windowing, demonstrating the improvement in image quality and focus over the course of iterations. As the aberration matrix detects the PSF within a window at a time, the focus gradually diminishes with each iteration while the Strehl ratio increases from 0.0045 to 0.12. Here, we increased the window size for outgoing fields to σ = 5 μm, which resulted in optimal performance.

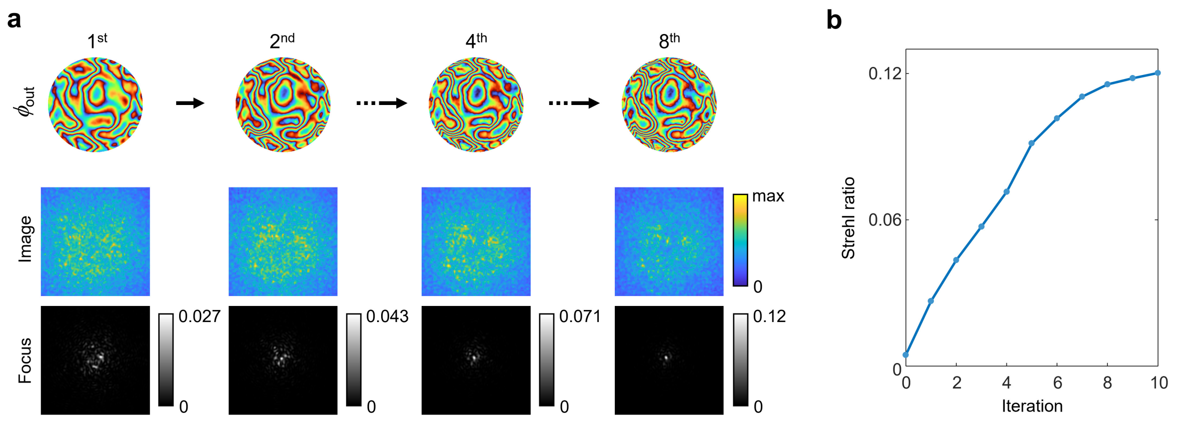

Supplementary Figure 2: Iterative windowing under strong surface aberration. a, The image quality and focus are enhanced at each iteration, as a result of the accumulation of outgoing aberration functions (ϕ_{out}) obtained from the aberration matrix. **b**, The Strehl ratio is presented as a function of the iteration number. Source data are provided as a Source Data file.

1.3. Application of the aberration matrix to experimental reflection matrix imaging data

The proposed approach can be applied to existing reflection matrix imaging (RMI) data by constructing the aberration matrix from the fields measured at adjacent illumination angle steps[1]. Figure S3 illustrates the results of our method applied to an RMI dataset of the human cornea, adapted from Balondrade et al[2,3]. As shown in Supplementary Fig. 3, our approach significantly enhances image quality compared to the uncorrected version and performs better than the existing method (explained in the main text) in terms of contrast, as highlighted in the orange circle of Supplementary Fig. 3.

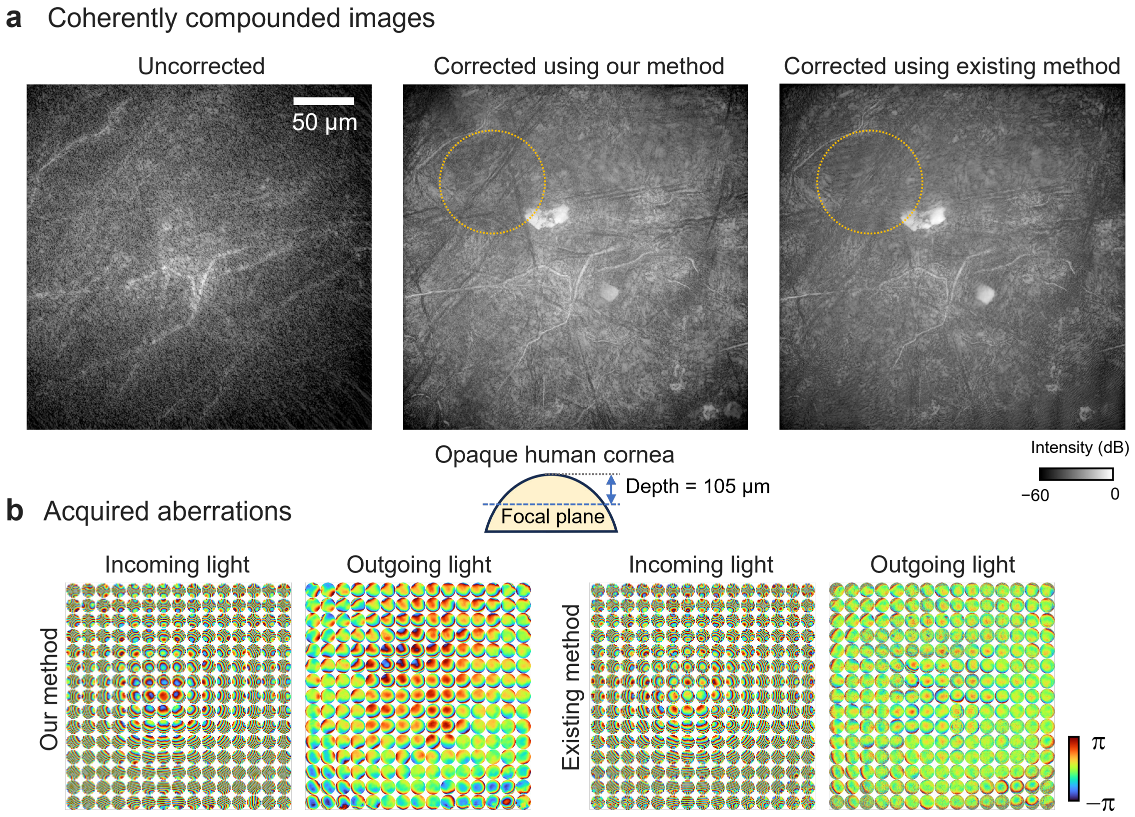

Supplementary Figure 3: Aberration correction in opaque human cornea. a, Coherently compounded images. Left: uncorrected, Middle: corrected using our method, Right: corrected using the existing method. **b**, Aberrations obtained from 15×15 points using our method (left), and the existing method (right). Experimental data was obtained from Zenodo[2].

2. Effect of the tilt angle on signal-to-noise ratio (SNR)

The quality of our method relies on the successful measurement of an aberration matrix. As elucidated in Eqs. (5)-(7), the phase of an aberration matrix exhibits the gradient of an aberration function as a signal, along with the decorrelation term serving as the error. In practical scenarios, measurement noise also contributes to the error to a considerable extent. We summarized the magnitudes of these three components in Supplementary Table 1.

Type	Component	Magnitude
Signal	Gradient of an aberration function	$\mathcal{O}(\Delta k)$
Error	Decorrelation term	$\mathcal{O}\left(\Delta k^2/k_{\mathrm{MER}}{}^2\right)$
	Measurement noise	$\mathcal{O}\left(\lvert T\rvert^{-2}\right)$

Supplementary Table 1 : Signal and error components in the phase of an aberration matrix.

It can be observed that the magnitudes of both signal and error components are dependent on the tilt angle (Δk). Consequently, the signal extent can be maximized by adjusting the tilt angle. To reduce the effect of the decorrelation term, it is necessary to keep the tilt angle below the memory effect range. At the same time, the tilt angle should be sufficiently large so that the desired signal is larger than the noise.

To further investigate the effect of the tilt angle, we present the central column of the aberration matrices, $A_{\Delta\mathbf{k}}(\mathbf{k}_{\mathrm{out}};\mathbf{k}_{\mathrm{in}}=\mathbf{0})$, constructed from fields scattered from the 100-μm thick tissue, in Supplementary Fig. 4. As the tilt angle increases, the signal, which is the gradient of the outgoing aberration function, becomes stronger. This effect is particularly notable in the polymer-added case compared to the other cases. At the same time, the phase become increasingly randomized, resulting in a diminished correlation, as illustrated in Fig. 6d. Based on these findings, the tilt angle was determined to be $\Delta k = 2\pi \times 0.0054/\lambda$, and it was fixed

throughout the experiments presented in the main text for convenience. Here, the aberration matrices were thresholded based on the angular spectrum of the fields ($|T|^2$) to discard the phases that were strongly affected by noise, as disscussed in Methods. The impact of the decorrelation term and noise can be further mitigated by measuring fields with a greater number of incident angles, thereby enhancing the SNR by a factor equal to the square root of the number of incident angles, in accordance with the central limit theorem.

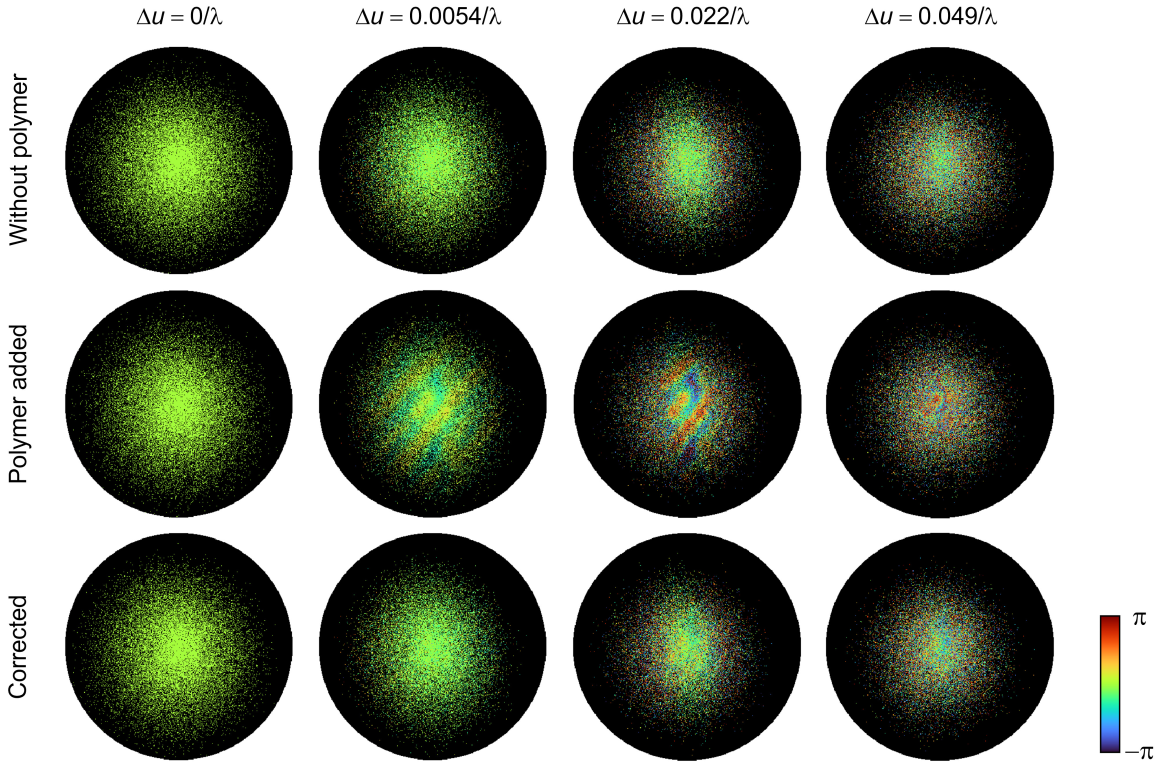

Supplementary Figure 4: Aberration matrices with different tilt angles. a, The central columns ($\boldsymbol{k}_{in}$=0) of the aberration matrices with different tilt angles. The aberration matrices were constructed from polymer-free, aberrated, and corrected fields scattered from the 100-μm thick tissue and then thresholded as described in Methods. Here, $\Delta\mathbf{u} = \Delta\mathbf{k}/2\pi$ is parallel to the horizontal direction.

3. Constructing the aberration matrix from the measured scattering matrix

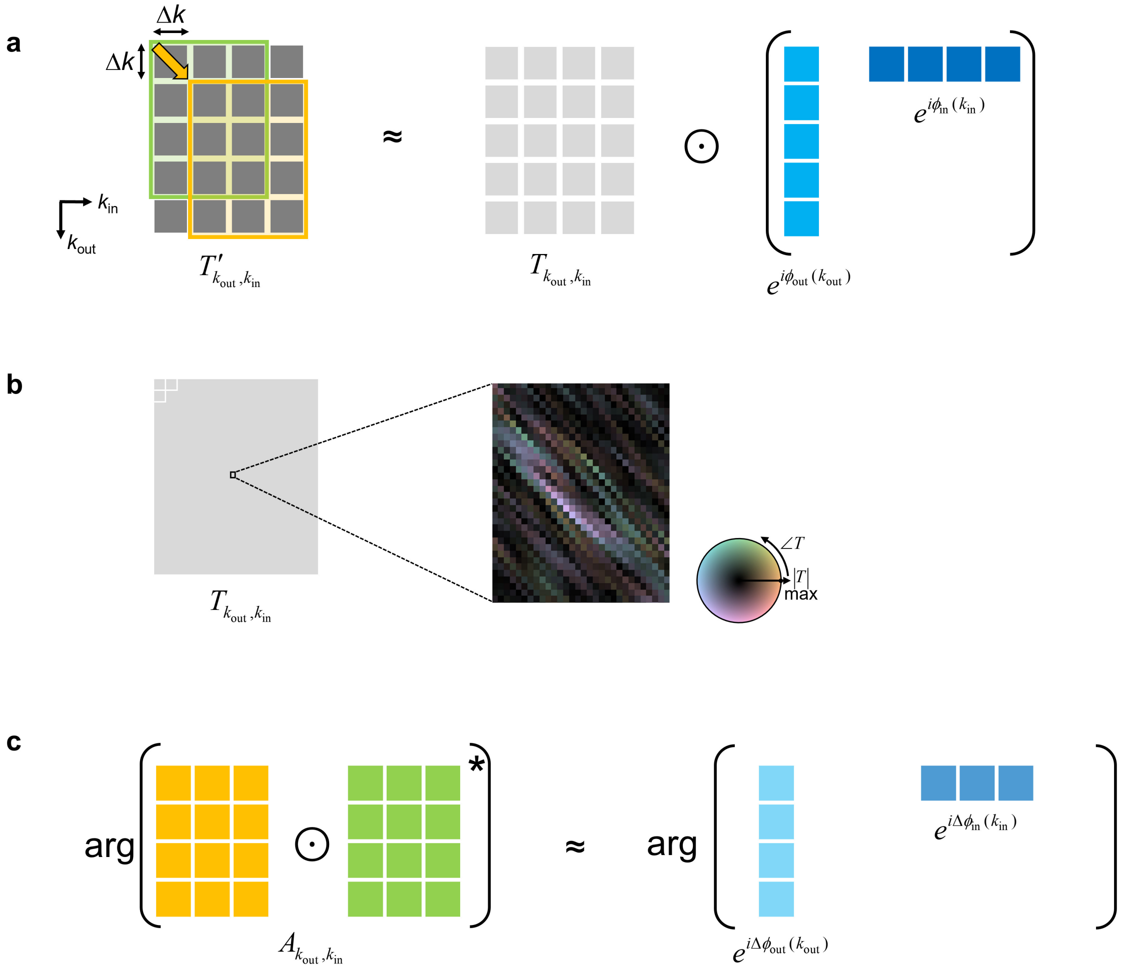

Supplementary Figure 5: Overview of the scattering matrix-based derivation. a, Visualization of Eq. (4) using scattering matrices in the k-space basis. T' represents the measured scattering matrix, while T represents the scattering matrix of the target object. ϕ_{out} and ϕ_{in} denote outgoing and incoming aberration function, respectively. The symbol $\odot$ denotes the element-wise multiplication. **b**, A Scattering matrix exhibiting the angular memory effect (Eq. (3)). A similar figure can be found in the study by Judkewitz et al[4]. **c**, Visualization of the aberration matrix exhibiting the aberration for outgoing and incoming light in its column and row, respectively. arg represents the element-wise argument of the matrix. The symbol * represents the complex conjugate.

Up to this point, we have derived and explained our method primarily from a 2D image processing perspective. In this section, we present a reformulation of our method using a scattering matrix, which can be either a transmission matrix or a reflection matrix. We begin by restating Eq. (4) in terms of the measured scattering matrix $T'_{k_{\text{out}},k_{\text{in}}}$ and the scattering matrix

of the target object $T_{k_{out},k_{in}}$ (Supplementary Fig. 5a):

$$T'_{k_{out},k_{in}} = T_{k_{out},k_{in}} \widetilde{P_{out}}(k_{out})\widetilde{P_{in}}(k_{in}), \tag{S1}$$

where $\widetilde{P_{out}}$ and $\widetilde{P_{in}}$ denote the column and row vectors describing the aberrations for outgoing and incoming light, respectively.

The anular memory effect states the there is a correlation along the diagonal direction within a scattering matrix. Thus, the following approximation holds if Δk is sufficiently smaller than the memory effect range:

$$T_{k_{out}+\Delta k,k_{in}+\Delta k} \approx T_{k_{out},k_{in}}. \tag{S2}$$

The anular memory effect can be directly observed from an unaberrated scattering matrix, as depicted in Supplementary Fig. 5b and a study conducted by Judkewitz et al[4]. Utilizing this correlation, it becomes possible to extract the aberration term from the measured scattering matrix as follows (Supplementary Fig. 5c):

$$\arg\left[T'_{k_{out}+\Delta k,k_{in}+\Delta k} \odot T'^{*}_{k_{out},k_{in}}\right] \cong \arg\left[\Delta\widetilde{P_{out}}(k_{out})\Delta\widetilde{P_{in}}(k_{in})\right], \tag{S3}$$

where we define $\Delta\widetilde{P_{out}}(k_{out}) = \widetilde{P_{out}}(k_{out}+\Delta k)\widetilde{P_{out}}^{*}(k_{out})$ and $\Delta\widetilde{P_{in}}(k_{in}) = \widetilde{P_{in}}(k_{in}+\Delta k)\widetilde{P_{in}}^{*}(k_{in})$, and used $\odot$ to denote the element-wise multiplication.

To utilize Eq. (S3), we define the aberration matrix,

$$A_{k_{out},k_{in}} = T'_{k_{out}+\Delta k,k_{in}+\Delta k} \odot T'^{*}_{k_{out},k_{in}}, \tag{S4}$$

from which we can obtain outgoing and incoming aberration functions using the matrix factorization method discussed in Methods.

4. Defocus and system-induced aberrations

In addition to sample-induced aberrations, defocus and system-induced aberrations are commonly encountered in real-world experiments. Defocus aberration occurs when the target volume is not centered in the focal plane (Supplementary Fig. 6a). In a time-gated microscope, such as optical coherence tomography (OCT), the defocus aberration can be characterized by the offset between the focal plane and the reference mirror (Supplementary Fig. 6b). Correcting the defocus aberration centers the target volume in the focal plane, which reduces the axial FOV and memory requirements for tomographic reconstruction.

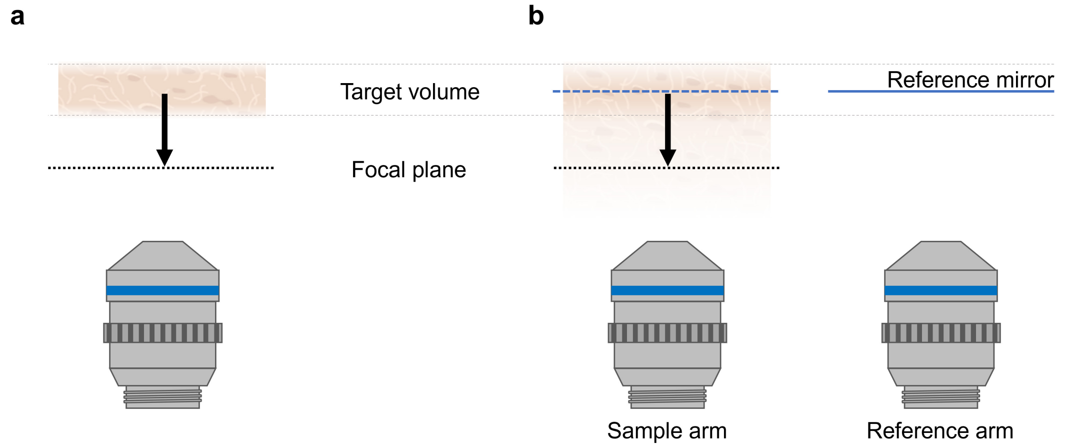

Supplementary Figure 6: Illustration of defocus aberration. a, Defocus aberration in a typical microscope. Target volume is out of the focal plane. **b**, Defocus aberration in a time-gated microscope (OCT). The focal plane and the reference mirror are not aligned. The amount of the defocus aberration is denoted by black arrows. Created with BioRender.com.

System-induced aberrations are an inevitable consequence in real optical systems due to the misalignment and imperfections of optical components. For example, tilt of the coverslip can

cause significant coma aberration in water-immersion objectives[5]. Consequently, the resulting tomograms suffer from blurring both laterally and axially, even when the polymer is not present.

To investigate these aberrations, we applied our method without placing the polymer layer. Specifically, we measured the fields of the 10-μm thick human tissue, which has minimal decorrelation effect among the samples we measured, at two random positions. Note that we increased the tilt angle (Δk) by a factor of 5 to more accurately characterize the aberrations.

As depicted in Supplementary Fig. 7, the aberrations detected from both positions manifest as defocus and system-induced aberrations, causing the reconstructed tomograms to be off-center in the z-direction and appear blurred. The defocus aberrations appear differently in the two positions, implying difficulty in focusing on the center of thick samples by eye. On the other hand, the system aberrations appear as a skewed doughnut shape in both positions. The doughnut shape is indicative of spherical aberrations, while the skewness indicates coma due to the tilt of the sample. While the shape of the system aberrations remained almost the same, they differed in the skew direction, suggesting a change in the tilt direction when the sample was moved. After correcting these aberrations, the tomograms were properly centered, accompanied by a notable enhancement in sharpness and contrast, particularly in the y-z and x-z sections.

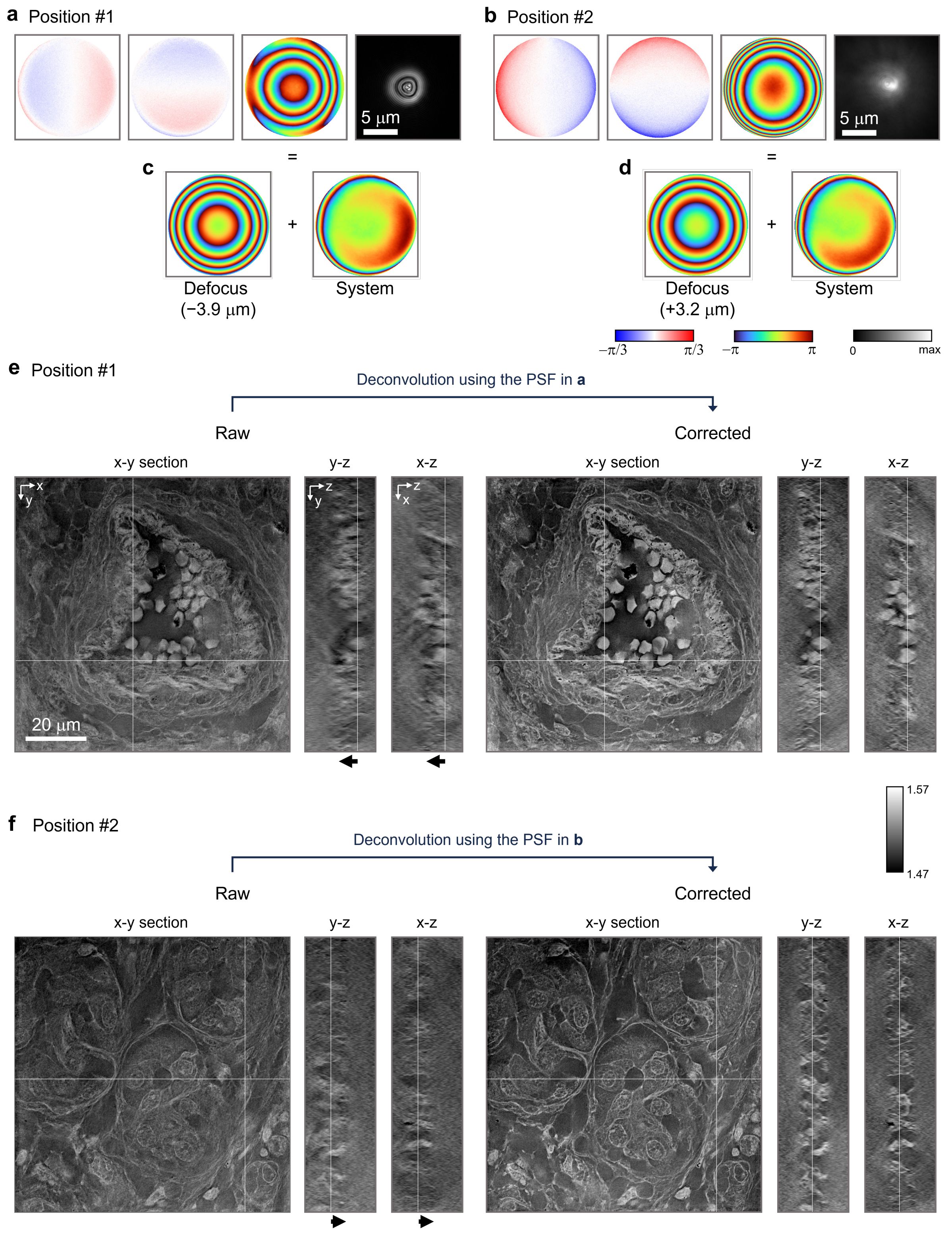

Supplementary Figure 7: Correction of defocus and system-induced aberrations with 10-μm thick human tissue. a–**b**, Aberrations detected at the first (**a**) and second (**b**) position of the samples: x- and y- components of the phase gradient, phase of the aberration function in *k*-space, and the corresponding PSF are shown from left to right, respectively. **c**–**d**, Decomposition of the aberration functions into defocus and other (denoted as system)

aberrations. **e**–**f**, Refractive index (RI) tomograms reconstructed using the measured (left) and corrected fields (right) at the first position (**e**) and second position (**f**). The z-axis is parallel to the optical axis.

5. Characteristics of noise from digital mirror device (DMD)

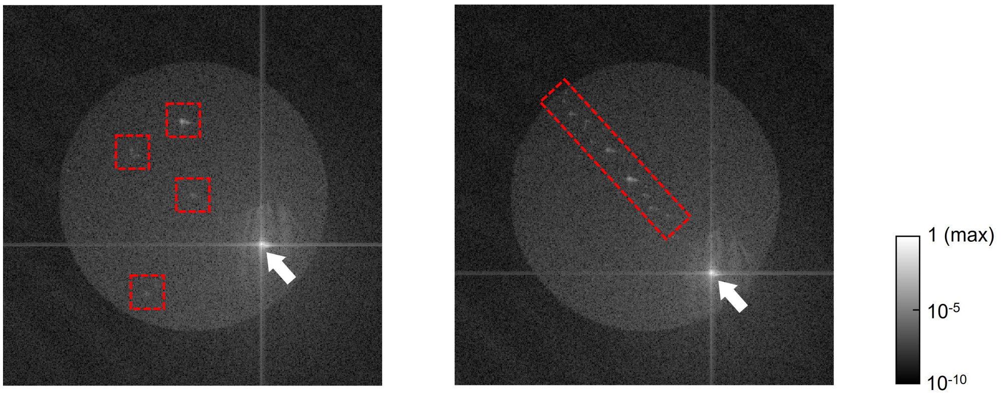

Supplementary Figure 8: DMD noise in plane wave illuminations. Fourier spectra of the two different plane waves are shown. DC peaks are denoted by white arrows, while peaky noise originating from the DMD is marked by red rectangles. The modulus of the Fourier spectra are shown in a log scale.

Since the DMD can only perform binary amplitude modulation (on and off), its capacity to manipulate arbitrary wavefronts is significantly restricted. In theory, this issue can be addressed by partitioning the desired amplitude into binary images and subsequently accumulating their interference patterns with a stationary reference within the camera's exposure time[6]. However, in practice, the temporal phase fluctuations between the reference and sample arm of an interferometer hinder the in-phase accumulation of interferograms. This results in small peaks generated by individual binary images, as shown in Supplementary Fig. 8. Notably, the DMD noise exhibits considerable variation even when the propagation angles of the two modulated plane waves are close to each other. This variability results in a reduced correlation between the scattered waves during the aberration detection process, consequently degrading the quality of the acquired aberration.

6. Additional analysis of aberration correction on 50-μm thick tissue

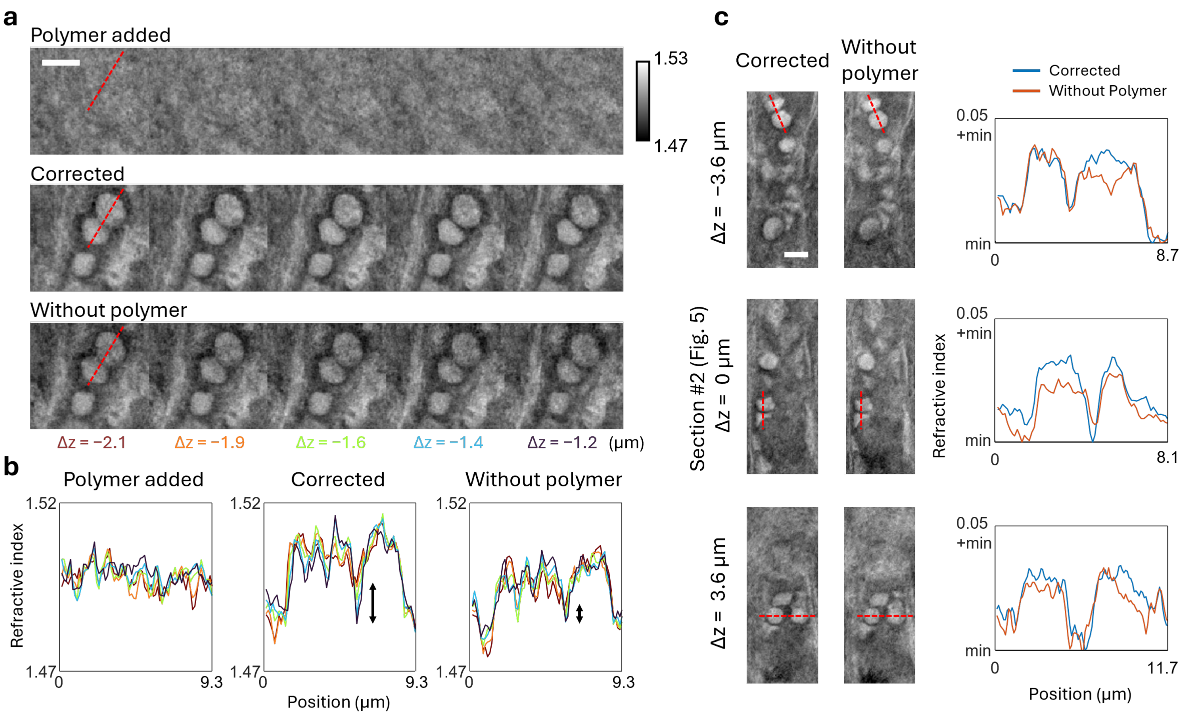

Supplementary Figure 9: Additional analysis of aberration correction on 50-μm-thick tissue. a, 3D refractive index (RI) stacks showing two red blood cells (RBCs) in contact at the initial plane, separating as the axial distance (Δz) increases. **b**, RI profiles along selected regions (red dashed line in **a**). The corrected tomogram reveals over twice the RI variation (gray arrow) compared to the polymer-free tomogram, as the gap between the RBCs widens. **c**, Comparison of corrected and polymer-free RI images of in-focus RBCs. The image profiles (right) show increased RI contrast in the corrected tomogram.

In Supplementary Fig. 9, we provide additional analysis of aberration correction in refractive index tomograms of 50-μm-thick tissue, focusing on the sharpness of red blood cells (RBCs). Figure S9a displays zoomed views of RBCs in three tomograms: polymer-added, corrected, and polymer-free. At the initial plane (Δz = -2.1 μm), the RBCs are in contact, gradually separating with increasing axial position. Figure S9b shows refractive index profiles taken along selected lines (red dashed lines in Supplementary Fig. 9a), where the corrected tomogram reveals a more than twofold increase in refractive index variation as the gap between RBCs

widens compared to the polymer-free tomogram. This suggests that aberration correction enhances both sharpness and refractive index contrast between neighboring structures. In Supplementary Fig. 9c, the refractive index images at various depths show the improved contrast for in-focus RBCs in corrected tomograms.

Supplementary References

1. Oh, C., Hugonnet, H., Lee, M. & Park, Y. GitHub. *https://github.com/BMOLKAIST/AberrationMatrix* (2024).
2. Balondrade, P. *et al.* Multi-Spectral Reflection Matrix for Ultra-Fast 3D Label-Free Microscopy. *Zenodo* (2023). https://doi.org:10.5281/zenodo.8407618
3. Balondrade, P. *et al.* Multi-spectral reflection matrix for ultrafast 3D label-free microscopy. *Nature Photonics* **18**, 1097-1104 (2024). https://doi.org:10.1038/s41566-024-01479-y
4. Judkewitz, B., Horstmeyer, R., Vellekoop, I. M., Papadopoulos, I. N. & Yang, C. H. Translation correlations in anisotropically scattering media. *Nat Phys* **11**, 684-689 (2015). https://doi.org:10.1038/Nphys3373
5. Arimoto, R. & Murray, J. M. A common aberration with water-immersion objective lenses. *J Microsc-Oxford* **216**, 49-51 (2004).
6. Lee, K., Kim, K., Kim, G., Shin, S. & Park, Y. Time-multiplexed structured illumination using a DMD for optical diffraction tomography. *Opt. Lett.* **42**, 999-1002 (2017). https://doi.org:10.1364/Ol.42.000999
